# Time in Brain: How Biological Rhythms Impact on EEG Signals and on EEG-Derived Brain Networks

**DOI:** 10.3389/fnetp.2021.755016

**Published:** 2021-09-27

**Authors:** Klaus Lehnertz, Thorsten Rings, Timo Bröhl

**Affiliations:** ^1^ Department of Epileptology, University of Bonn Medical Centre, Bonn, Germany; ^2^ Helmholtz Institute for Radiation and Nuclear Physics, University of Bonn, Bonn, Germany; ^3^ Interdisciplinary Center for Complex Systems, University of Bonn, Bonn, Germany

**Keywords:** electroencephalography, biological rhythms, brain dynamics, statistical moments, synchronization, functional brain networks, clustering coefficient, centrality

## Abstract

Electroencephalography (EEG) is a widely employed tool for exploring brain dynamics and is used extensively in various domains, ranging from clinical diagnosis via neuroscience, cognitive science, cognitive psychology, psychophysiology, neuromarketing, neurolinguistics, and pharmacology to research on brain computer interfaces. EEG is the only technique that enables the continuous recording of brain dynamics over periods of time that range from a few seconds to hours and days and beyond. When taking long-term recordings, various endogenous and exogenous biological rhythms may impinge on characteristics of EEG signals. While the impact of the circadian rhythm and of ultradian rhythms on spectral characteristics of EEG signals has been investigated for more than half a century, only little is known on how biological rhythms influence characteristics of brain dynamics assessed with modern EEG analysis techniques. At the example of multiday, multichannel non-invasive and invasive EEG recordings, we here discuss the impact of biological rhythms on temporal changes of various characteristics of human brain dynamics: higher-order statistical moments and interaction properties of multichannel EEG signals as well as local and global characteristics of EEG-derived evolving functional brain networks. Our findings emphasize the need to take into account the impact of biological rhythms in order to avoid erroneous statements about brain dynamics and about evolving functional brain networks.

## 1 Introduction

The human brain is an open, dissipative, and adaptive dynamical system that can be described as a complex network of networks of interacting subsystems. It is an inherently nonstationary system, whose complicated spatial-temporal dynamics is still poorly understood. In order to gain deeper insights, various measurement techniques are employed to record—on different spatial scales and with different levels of invasiveness—time series of observables related to e.g. electric and/or magnetic fields or thermodynamic and chemical properties. Among these measurement techniques, electroencephalography (EEG) is the only technique that allows for the continuous multichannel recording of time series of macro-scale brain dynamics over extended periods of time (days to weeks and beyond (Niedermeyer and Lopes da Silva, 2005; Weisdorf et al., 2019; Viana et al., 2021)). In case of brain pathologies (such as epilepsy), invasive electroencephalography provides additional access to the meso- (≈10^5^ neurons) and the micro-scale (single neurons) (Engel et al., 2005; Chang, 2015). Electroencephalography allows to capture a wide spectrum of physiological and pathophysiological activities on various time scales.

Access to brain dynamics using EEG can be gained through active perturbations or passive observations. In the first case, evoked or event-related potentials (EP/ERP) are assumed to reflect the synchronized neuronal relaxation dynamics of specific brain regions elicited by motor, sensory, or cognitive tasks. On a more global level, cortical excitability can be probed with e.g. electrical or magnetic stimulation (Hallett, 2007; Yang et al., 2021). With these approaches, the recorded relaxation dynamics is typically confined to time scales ranging from a few milliseconds to a few seconds. In the second case, ongoing (i.e., non-triggered) EEG signals are recorded e.g. during sleep, states of wakefulness (daily life activities), or during specific neuropsychological tasks that control sensory inputs and/or higher cognitive functions. Such recordings capture brain dynamics on time scales that range from a few seconds to hours and days and beyond. Both these cases require special time series analysis techniques. Modern EEG analysis techniques allow investigation of various linear and nonlinear aspects of ongoing brain dynamics of single brain regions as well as of properties of interactions (strength, direction, coupling functions) between the dynamics of two or more brain regions. Together with graph-theoretical concepts these analysis techniques provide a means to characterize the dynamical evolution of brain networks (for an overview, see e.g. Niedermeyer and Lopes da Silva (2005), Pereda et al. (2005), Stam (2005), Lehnertz et al. (2009), Lehnertz (2011), Sakkalis (2011), Greenblatt et al. (2012), Lehnertz et al. (2014), Lehnertz et al. (2017).

**FIGURE 1:**
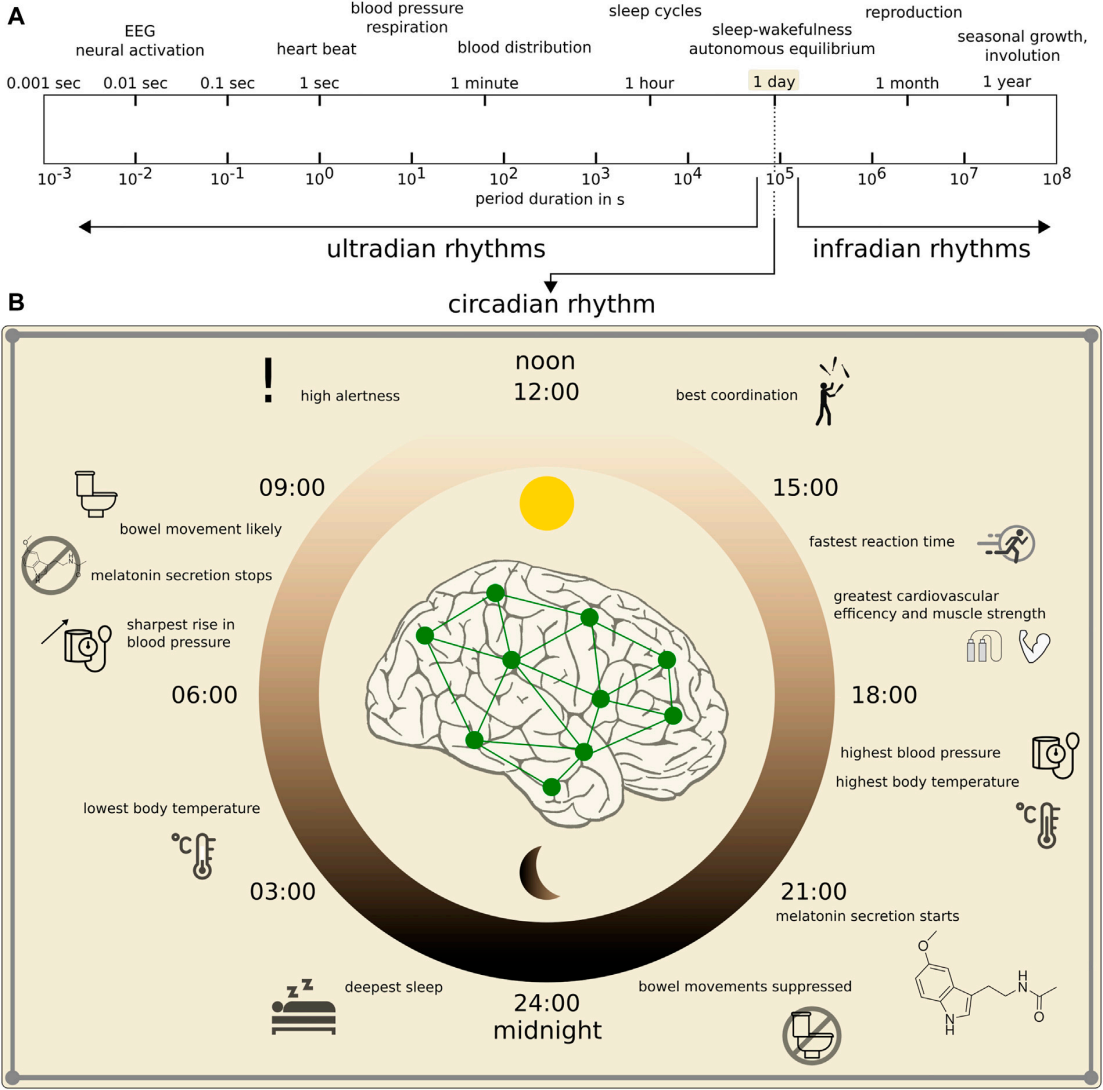
Spectrum of main biological rhythms in humans. **(A)**: Logarithmic presentation of period durations of rhythms (modified after Hildebrandt (1991)). **(B)**: Zoom into human circadian rhythm with some behavioral and physiological functions within the 24 h cycle that impact on the dynamics of the brain and other organ systems.

Like many other physiologic observables, EEG signals are influenced by various endogenous and exogenous biological rhythms (Aschoff, 1981; Rensing et al., 1987). Among these rhythms, the circadian rhythm—a roughly 24-h cycle (range: 20—28 h; see Figure 1)—is probably the best investigated rhythm (Mills, 1966; Halberg, 1969; Folkard et al., 1983; Spengler et al., 2000; Cermakian and Boivin, 2003; Bell-Pedersen et al., 2005; Iskra-Golec, 2006; Czeisler and Gooley, 2007; Franken and Dijk, 2009; Mohawk et al., 2012; Gillette, 2013; Ly et al., 2016; Duboc et al., 2020; Foster, 2020; Garbarino et al., 2020; Hablitz et al., 2020; Lananna and Musiek, 2020; Matenchuk et al., 2020). Ultradian rhythms have shorter periods than the circadian rhythm’s period, and periods are often defined to be shorter than 20 h but longer than 1 h. These rhythms are often not directly related to environment cycles which renders their interpretation difficult. Prominent examples include the 90—120 min cycling of the sleep stages (Dement and Kleitman, 1957) and the basic rest-activity cycle (Klein and Armitage, 1979; Lavie and Kripke, 1981; Kleitman, 1982). Infradian rhythms have longer periods than the circadian rhythm’s period, i.e., longer than 28 h. Prominent examples include the circaseptan rhythm (weekly rhythm, 7 ± 3 days), the circatrigintan rhythm (monthly rhythm, 30 ± 5 days, e.g. menstruation), and the circannual rhythm (yearly rhythm, 1 year ±2 months). Interactions between the various rhythms are not fully understood (Laje et al., 2018).

The impact of particularly the circadian rhythm and of ultradian rhythms on EEG signals is known for more than 50 years (for circadian rhythms, see, e.g. Frank et al. (1966); Gundel and Witthöft (1983); Machleidt (1980); Borbély et al. (1981); Torsvall and Åkerstedt (1987); Cacot et al. (1995); Borbély and Achermann (1999); Aeschbach et al. (1999); Dijk and Duffy (1999); for ultradian rhythms, see, e.g. Manseau and Broughton (1984); Oken and Chiappa (1988); Tsuji and Kobayashi (1988); Hayashi et al. (1994); Kaiser (2008); Piarulli et al. (2016)). Many seminal studies, however, were based on EEG recordings that covered time periods ranging from a few seconds to a few hours and/or captured the dynamics of only a few brain regions. Moreover, most studies concentrated on the rhythms’ impact on spectral characteristics of EEG signals, i.e., on changes of spectral power in the well-known alpha-, beta-, theta-, and delta-frequency band. Given recent technological developments that enable ultra-long (days to weeks and beyond) scalp (Casson, 2019), sub-scalp (Weisdorf et al., 2019; Duun-Henriksen et al., 2020), and intracortical (Zaer et al., 2021) EEG recordings in diverse applications (Aricò et al., 2018; Abiri et al., 2019; Cinel et al., 2019; Lohani et al., 2019; Alsuradi et al., 2020; Dehais et al., 2020; Rashid et al., 2020) even beyond clinical ones, it is important to understand the impact of endogenous and exogenous rhythms on characteristics of brain dynamics assessed with the aforementioned modern EEG analysis techniques.

## 2 From Local to Global: Impact of Biological Rhythms

In the following, we highlight some important aspects of this impact by discussing findings that we obtained from analyses of exemplary continuous multiday, multichannel EEG signals recorded non-invasively and invasively from two subjects. We here concentrate on exemplary characteristics of brain dynamics and of so called functional brain networks (Bullmore and Sporns, 2009) that we estimated from EEG signals with various analysis techniques using a moving-window approach (window length: 20 s; non-overlapping window; demeaned artifact-free data). The chosen window length can be regarded as a compromise between the required statistical accuracy for the calculation of the various characteristics and approximate stationarity of EEG signals within a window’s duration (Isaksson et al., 1981; Blanco et al., 1995; Rieke et al., 2003).

For each window and each sampled brain region, we estimated the respective dynamics’ statistical moments (Press et al., 2007): standard deviation *σ*, skewness *s*, and (excess) kurtosis *k*. For each window and each (non-redundant) pair of sampled brain regions, we characterized their strength of interaction employing a phase-based (mean phase coherence *R* (Mormann et al., 2000)) and an amplitude-based estimator (absolute value of the linear correlation coefficient *ρ* (Press et al., 2007)). Both these estimators are often used to derive a functional brain network, whose vertices are associated with the sampled brain regions and whose edges represent the strength of interaction between pairs of vertices (often referred to as functional connectivity; see Bastos and Schoffelen (2016) for an overview). We here proceeded in that way and estimated the network’s clustering coefficient *C* ^X^ as well as eigenvector centrality for vertices $C_{\text{v}}^{\text{X}}$ and for edges $C_{\text{e}}^{\text{X}}$ (Lehnertz et al., 2014; Bröhl and Lehnertz, 2019), where the superscript X is a placeholder for the employed estimator for the strength of interaction (*R* or *ρ*). This resulted in a temporal sequence of various EEG characteristics and of characteristics of EEG-derived, fully connected, weighted and undirected, functional brain networks. We then adopted a pragmatic approach to investigate the contribution of timescales of endogenous and exogenous rhythms on the characteristics’ temporal variability and estimated the power spectral densities (Lomb-Scargle periodogram (Press and Rybicki, 1989)) of the respective time series (other analysis tools (Huang et al., 1998; Kantelhardt et al., 2001) might be better suited for nonstationary data).

### 2.1 Impact on Temporal Changes of Dynamics’ Characteristics of Single Brain Regions

**FIGURE 2:**
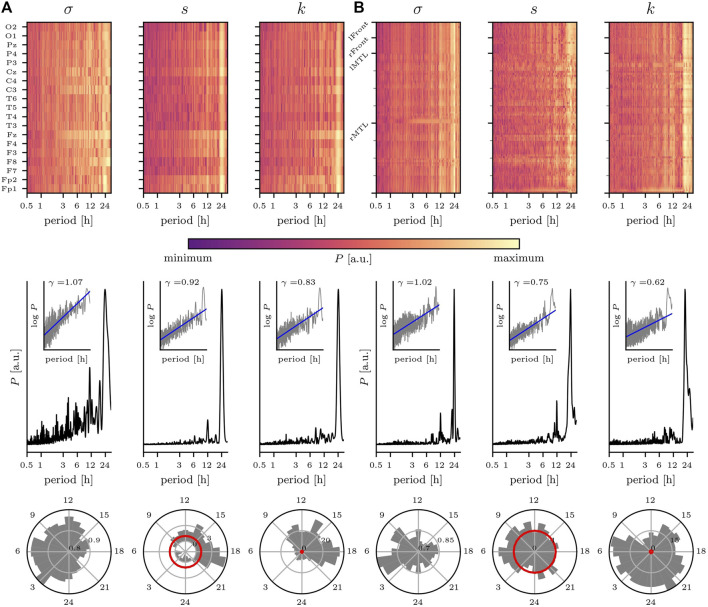
Impact of circadian rhythm and of ultradian rhythms on temporal changes of statistical moments of brain dynamics. Exemplary findings for **(A)** non-invasive EEG (nEEG) recording lasting 7 days (recording sites shown on *y*-axis; data from a male subject (81 y) with cognitive impairment under CNS drugs admitted for evaluation of epilepsy risk) and **(B)** intracranial EEG (iEEG) recording lasting 14 days (sampled brain regions (left/right mesial temporal lobe (MTL) and left/right frontal areas) shown on *y*-axis; data from a male subject (55 y) with epilepsy under CNS drugs admitted for presurgical evaluation). Top: relative power spectral densities (*P*; color coded) of time series of standard deviation *σ*, skewness *s*, and kurtosis *k* from each recording site. Middle: averaged relative power spectral densities (mean over all sampled brain regions). Insets show log-log plots of data (grey) together with linear least squares lines (blue, log  *P* = *γ* log  *π*, where *π* denotes period (range: 30 min to 32 h) and *γ* denotes the scaling exponent). Bottom: circadian distribution of statistical moments (24 h bins; mean over all sampled brain regions). Note that for Gaussian distributed data, skewness and (excess) kurtosis would be zero with their respective standard deviation indicated by the red circle. For *σ*, the outermost circle indicates the maximum value and inner circles the relative percentage. For *s* and *k*, the grey/black circles indicate the factor by which data deviates from the standard deviation of Gaussian distributed data (red circle). EEG data sampled at 256 Hz **(A)** 250 Hz **(B)**; 16 bit ADC; bandwidth 1—45 Hz; notch filter at line frequency (50 Hz).

We begin with discussing the impact of various rhythms on the temporal changes of statistical moments estimated for the dynamics of 19 brain regions recorded non-invasively with scalp EEG (nEEG) (Niedermeyer and Lopes da Silva, 2005) (Figure 2A). A large fraction of the temporal variability of the nEEG signals’ standard deviation, skewness, and kurtosis can be attributed to the circadian rhythm with a period length at about 24 h. Interestingly, both the rhythm’s intensity and its period length vary for the different recording sites, which would indicate that the dynamics of the different brain regions are differently affected by this rhythm. Similar observations can be made for ultradian rhythms with period lengths at about 12, 8, 6, 4 h, 90, and 60 min but with comparably smaller intensities. Topographically, these rhythms are most pronounced in fronto-central areas, and these areas are known to reflect, for instance, the dynamics of the wake-dependent, or homeostatic component of sleep regulation (Cajochen et al., 2002; Croce et al., 2018). Another important aspect of the rhythms’ impact can be identified from the circadian distribution of nEEG signals’ statistical moments. Within the circadian cycle, the standard deviation attains highest values during the nighttime, as expected (Hjorth, 1970, 1973). Notably, the nEEG signals’ third and fourth statistical moment indicate a clear deviation from Gaussianity for data recorded during the daytime.

We observe a similarly pronounced impact of the circadian rhythm on statistical moments of intracranially recorded brain dynamics (Figure 2B). Remarkably, for the iEEG signals’ standard deviation *σ*, the 24 h peak in the corresponding periodogram is rather narrow, and one might speculate that this can be related to the narrow spatial sampling of circumscribed brain regions. Apart from rhythms with period lengths at about 12, 8 and 4 h, other ultradian rhythms contribute only to a small extent to the temporal variability of the iEEG signals’ statistical moments. Within the circadian cycle, the standard deviation again attains highest values during the nighttime, while skewness and kurtosis here indicate a clear deviation from Gaussianity mostly independent of the time of day.

It is important to note that the observed deviations from Gaussianity—seen for both nEEG and iEEG signals and that differentially depend on the time of day—may strongly impact on both the design of EEG-based investigations of various physiological and pathophysiological phenomena and the suitability and reliability of various EEG analysis techniques that assume the data to be Gaussian distributed. In the same manner, the observed 1/*f*-like temporal fluctuations of the EEG signals’ statistical moments (as indicated by the scaling exponents in the range $\gamma \in \left[0.6,1.1\right]$ ) not only contributes critically to the ongoing debate on the functional significance of scale-free brain dynamics (Bédard et al., 2006; Chialvo, 2010; Beggs and Timme, 2012; Papo, 2013; He, 2014; Papo, 2014; Lehnertz et al., 2017; Uddin, 2020) but also can be regarded a cautionary tale for EEG-based studies of e.g. cycles in epilepsy (Karoly et al., 2018; Baud et al., 2021; Leguia et al., 2021) or of seizure-precursor identification using e.g. the concept of critical slowing down (see Wilkat et al. (2019) and Hagemann et al. (2021) and references therein for a critique). Not taking into account dependencies of statistical characteristics of EEG signals on biological rhythms can lead to erroneous statements about brain dynamics.

### 2.2 Impact on Temporal Changes of Interactions Between Brain Regions

**FIGURE 3:**
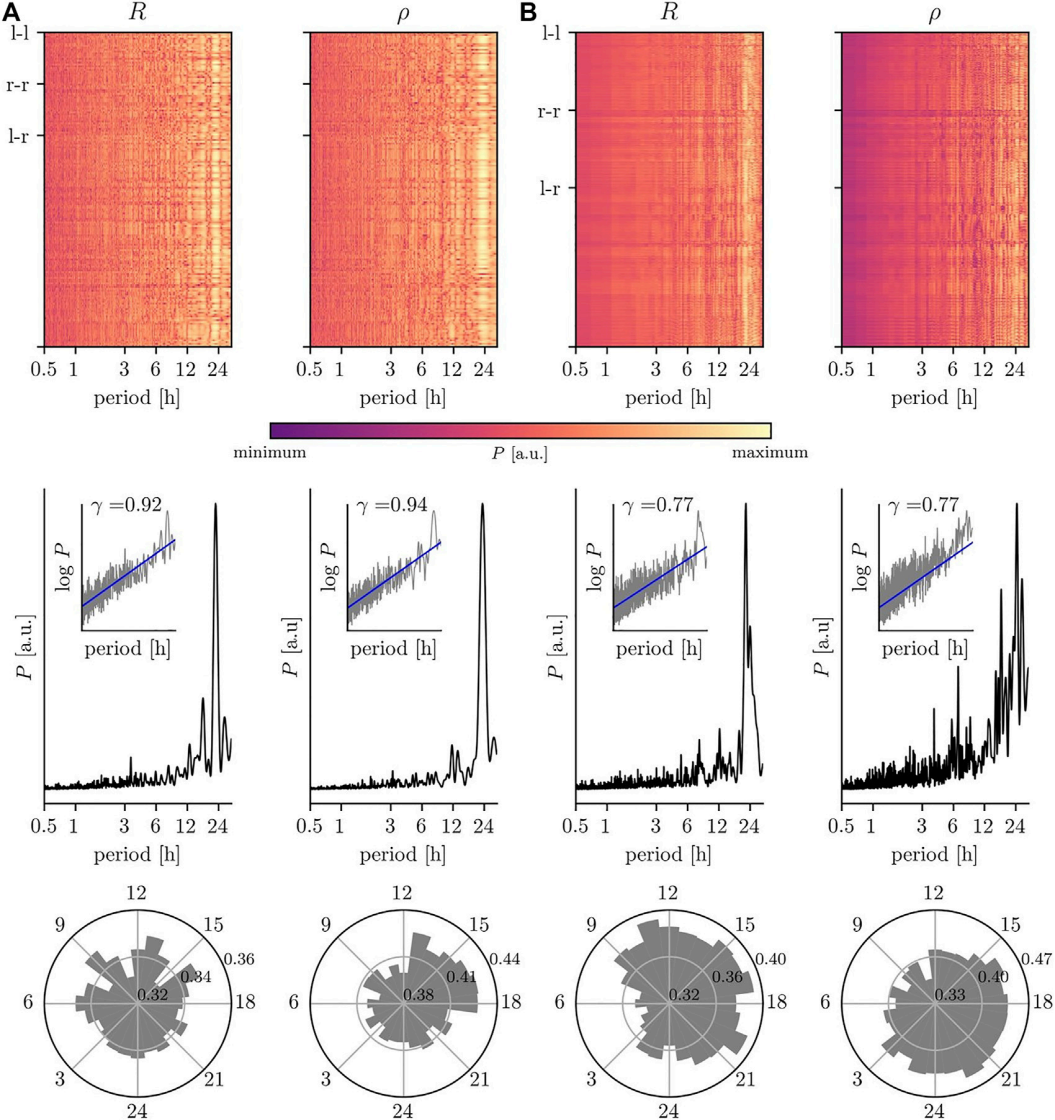
Impact of circadian rhythm and of ultradian rhythms on temporal changes of interaction properties of brain dynamics. Same EEG data as in Figure 2: **(A)** scalp recording; **(B)** intracranial recording. **Top:** relative power spectral densities (color coded) of time series of estimates of the strength of interaction between each (non-redundant) pair of sampled brain regions (estimates based on mean phase coherence *R* and on linear correlation coefficient *ρ*). Intrahemispheric interactions are labeled l–l/r–r and interhemispheric interactions l–r. **Middle:** averaged relative power spectral densities (mean over all non-redundant pairs of sampled brain regions). Insets show log-log plots of data (grey) together with linear least squares lines (blue, see Figure 2 for details). **Bottom:** circadian distribution of estimated interaction properties (24 h bins; mean over all non-redundant pairs of sampled brain regions).

We proceed with discussing the impact of the various biological rhythms on the temporal changes of interactions between the dynamics of the sampled brain regions (Figure 3). For both, the phase-based and the amplitude-based estimator for the strength of interaction and independent of the type of recording, we observe—on average—a pronounced impact of the circadian rhythm. Note, however, that interactions are not equally affected by this rhythm; its impact may vary for short-ranged (nearest neighbor brain regions), intermediate-ranged (regions within same hemisphere), and long-ranged (across hemispheres) interactions (cf. Kreuz et al. (2004) and Lehnertz et al. (2017)). Moreover, for some of these interactions we observe additional contributions with period lengths around 17–19 h and around 27–30 h, which lead to either a triplett-like structure together with the circadian peak or to a broadening of that peak in the averaged periodogram.

Different ultradian rhythms appear to impact on temporal changes of the strength of interactions estimated from nEEG signals (Figure 3A) and from iEEG signals (Figure 3B) but with comparably smaller intensities. For the former, we observe contributions at period lengths around 3.5 and 12 h, while for the latter period lengths around 5 and 8 h or around 4 and 7 h can be identified depending on the employed estimator for the strength of interactions (*R* or *ρ*) together with an additional contribution at 12 h (cf. Porz et al. (2014)). A differential impact of these biological rhythms on the strength of interactions, estimated with either *R* or with *ρ*, can also be observed within the circadian cycle. Independent of the type of recording, the strengths of interactions are comparably higher during the daytime, which would point to a decreased level of synchronization between brain regions during the nighttime (Steriade and Hobson, 1976; Horovitz et al., 2009; Lazar et al., 2015; Mizrahi-Kliger et al., 2018; Nguyen et al., 2018). Interestingly, the daytime amplitude-based contributions (estimated with *ρ*) appear to be delayed by 3–6 h to the phase-based contributions (estimated with *R*). We can not yet provide an explanation for such a time delay, but it would need to be taken into account in comparative studies of e.g. functional connectivity or (patho-)physiologic synchronization phenomena.

In line with a number of previous studies (see, e.g., Gong et al. (2003); Stam and De Bruin (2004); Botcharova et al. (2014); Racz et al. (2018); Daffertshofer et al. (2018); Stylianou et al. (2020); La Rocca et al. (2021)), we observe 1/*f*-like temporal fluctuations of characteristics of brain interactions (as indicated by the scaling exponents $\gamma \in \left[0.77,0.94\right]$ ). We conjecture that such scale-free-like brain interaction properties can be traced back to specific aspects of the dynamics of brain regions involved in these interactions. We also conjecture that the various biological rhythms impact in a similar manner on the other properties of interactions, namely direction and coupling functions. Not taking into account dependencies of interaction properties on biological rhythms can lead to erroneous statements about brain interactions. At the same token, such dependencies may rank among the main determinants of the repeatedly reported low reproducibility of functional brain imaging studies (e.g. (Calhoun et al., 2014; Hodkinson et al., 2014; Zalesky et al., 2014; Nakamura et al., 2015; Open Science Collaboration, 2015; Preti et al., 2017; Thomas et al., 2018; Facer-Childs et al., 2019; Lurie et al., 2020; Orban et al., 2020; Specht, 2020)).

### 2.3 Impact on Temporal Changes of Characteristics of Evolving Functional Brain Networks

**FIGURE 4:**
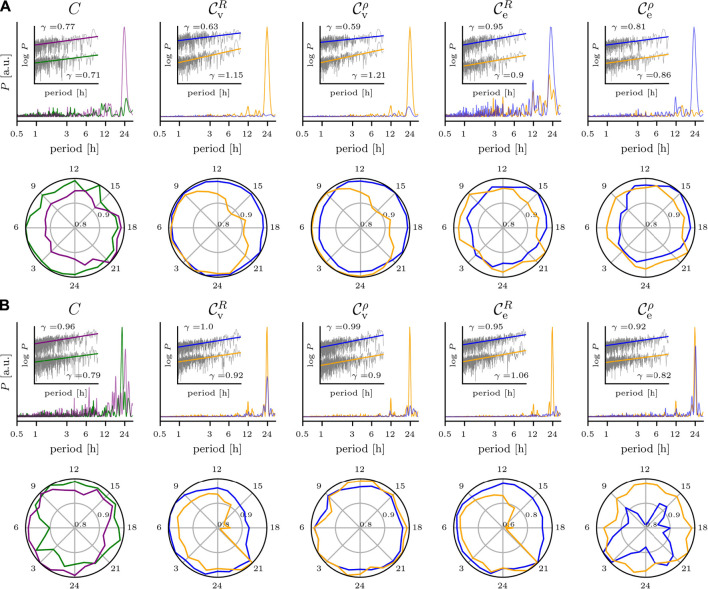
Impact of circadian rhythm and of ultradian rhythms on temporal changes of global and local characteristics of evolving brain networks. Same EEG data as in Figure 2. **(A)** Scalp recording; upper row, from left to right: relative power spectral densities of time series of the networks’ clustering coefficient *C* (derivation of network edges based on mean phase coherence *R* (green) or on linear correlation coefficient *ρ* (purple)), of eigenvector centralities $C_{\text{v}}^{\text{X}}$ of selected vertices (central (orange); occipital (blue)), and of eigenvector centralities $C_{\text{e}}^{\text{X}}$ of selected edges (central—occipital (orange), occipital—occipital (blue); X refers to employed estimator (*R* or *ρ*) for derivation of network edges). Insets show log-log plots of data (grey) together with linear least squares lines (see Figure 2 for details; data shifted to enhance readability). Lower row: circadian distribution of estimated network characteristics (24 h bins; colors as is upper row). The outermost circle indicates the maximum value and inner circles the relative percentage. **(B)** Intracranial recording; upper row, same as in **(A)**, but for selected vertices from right mesial temporal lobe (RMTL; orange) and from left frontal area (LF, blue) and for selected edges (RMTL—LF (orange); LF—LF (blue). Lower row as in **(A)**.

Eventually, we discuss the impact of the various biological rhythms on temporal changes of exemplary global (clustering coefficient (*C* ^*R*^ and *C* ^*ρ*^)) and local characteristics (centralities of vertices ( $C_{\text{v}}^{R}$ and $C_{\text{v}}^{\rho }$ ) and edges ( $C_{\text{e}}^{R}$ and $C_{\text{e}}^{\rho }$ )) of evolving brain networks (Figure 4). In general, we observe for both EEG recordings a vastly different impact of biological rhythms on network characteristics. Importantly, the impact strongly depends on which estimator (*R* or *ρ*) was employed to derive network edges.

### Clustering Coefficient

The temporal changes of *C* ^*ρ*^ of nEEG-based evolving networks (Figure 4A) are strongly dominated by the circadian rhythm with only minor contributions from ultradian rhythms with period lengths ranging from 1 h to about 14 h. In contrast, for the same networks but with edges derived via *R*, the circadian rhythm’s impact on *C* ^*R*^ compares to the ones seen for the aforementioned ultradian rhythms. Within the circadian cycle, fluctuations of both *C* ^*R*^ and *C* ^*ρ*^ amount to a few percent.

For the clustering coefficient of iEEG-based evolving networks (Figure 4B), the dependence of the rhythms’ impact on the estimator used to derive edges is less pronounced. The circadian rhythm appears to be decomposed into quartett-like structures with contributions at period lengths 22, 24, 19, and 27 h (descending order of intensities) for *C* ^*R*^ and at 25, 28, 21, and 19 h for *C* ^*ρ*^. For *C* ^*R*^, we observe contributions of ultradian rhythms with period lengths at about 15, 12, 8, and 3 h but with comparably small intensities. For *C* ^*ρ*^, there are contributions with higher intensities and with period lengths at about 17.5, 15, and 16 h (triplett-like structure) as well as at 13, 8.5, 6.5, and 3.8 h. As with the nEEG-based evolving networks, fluctuations within the circadian cycle of clustering coefficients amount to a few percent. Comparable findings were reported for the clustering coefficient as well as for other global characteristics of binary evolving brain networks derived from continuous, long-term, multichannel iEEG (Kuhnert et al., 2010; Geier et al., 2015; Lehnertz et al., 2017) and nEEG data (Mitsis et al., 2020) recorded from a larger group of epilepsy patients. Note that the temporal fluctuations of the clustering coefficients of the nEEG-based and iEEG-based evolving brain networks comply with a power-law with a scaling exponent *γ* in the range $\gamma \in \left[0.71,0.96\right]$ ). We are not aware of previous reports on such an observation.

### Vertex and Edge Centralities

For the nEEG-based evolving networks (Figure 4A), we concentrate on centrality ( $C_{\text{v}}^{R}$ and $C_{\text{v}}^{\rho }$ ) of vertices associated with a central brain area (cf. Section 2.1) and with occipital brain areas (Kuhnert et al., 2012) as well as on centrality ( $C_{\text{e}}^{R}$ and $C_{\text{e}}^{\rho }$ ) of edges connecting these brain areas (central—occipital and left occipital—right occipital). Using a centrality-based ranking (Liao et al., 2017) and independent of the estimator employed to derive network edges, we find the occipital vertices to rank higher, on average, than the central vertex. The same holds true for the edge connecting the occipital vertices when compared to the edge connecting the occiptal and the central vertex. Note, that these high-ranking vertices and edges are not the most important (highest rank) network constituents. We observe a strongly pronounced impact of the circadian rhythm on temporal changes of centrality ( $C_{\text{v}}^{R}$ and $C_{\text{v}}^{\rho }$ ) of the central vertex (which was to be expected, at least to some extent, from our findings in Section 2.1). Apart from a comparably small contribution with a period length at around 12 h, the impact of other ultradian rhythms appears negligible. For temporal changes of centrality of the occipital vertex, however, the rhythms’ impact is strongly reduced ( $C_{\text{v}}^{\rho }$ ) or barely detectable ( $C_{\text{v}}^{R}$ ) (note the reduced range of the scaling exponent $\gamma \in \left[0.59,0.63\right]$ ). The differential impact of the biological rhythms on vertex centralities is also reflected within the circadian cycle. We only observe during the daytime substantial alterations (∼ 10*%*) of the otherwise almost constant centrality of the central vertex. Constancy is also seen for the centrality of the occipital vertex.

Now, for temporal changes of edge centrality ( $C_{\text{e}}^{R}$ and $C_{\text{e}}^{\rho }$ ), it is surprisingly the edge connecting the occipital vertices for which we observe a strong impact of the circadian rhythm. For the other edge (that connects central and occipital vertices), the dependence of the rhythms’ impact on the estimator used to derive network edges is strongly pronounced. For $C_{\text{e}}^{\rho }$ , the rhythms’ impact is barely detectable, and for $C_{\text{e}}^{R}$ , we observe contributions with period lengths at 18, 22, and 25 h as triplett-like structures. In addition to comparably strong contributions with a period length at around 12 h, we observe contributions from other ultradian rhythms down to period lengths around 60 min, particularly for temporal changes of $C_{\text{e}}^{R}$ for both investigated edges. Within the circadian cycle, the fluctuations of edge centralities associated with these rhythms amount to a few percent.

For the iEEG-based evolving networks (Figure 4B), we concentrate on centrality ( $C_{\text{v}}^{R}$ and $C_{\text{v}}^{\rho }$ ) of vertices associated with the right mesial temporal lobe (RMTL) and with the left frontal (LF) area (see Section 2.1) as well as on centrality ( $C_{\text{e}}^{R}$ and $C_{\text{e}}^{\rho }$ ) of edges connecting these brain areas (RMTL—LF and LF—LF). As with the nEEG-based evolving networks, we use a centrality-based ranking to estimate the importance of the chosen vertices and edges. Independent of the estimator employed to derive network edges, we find the RMTL vertex to rank higher, on average, than the LF vertices, and the same holds true for the edge connecting the LF vertices. We also note here, that these high-ranking vertices and edges are not the most important (highest rank) network constituents. Independent of the estimator employed to derive network edges, we observe a strongly pronounced impact of the circadian rhythm on temporal changes of centrality ( $C_{\text{v}}^{R}$ and $C_{\text{v}}^{\rho }$ ) of the RMTL vertex. For $C_{\text{v}}^{R}$ , we find a triplett-like structure with a period length of a main peak at around 24 h as well as less pronounced contributions at 21 and 29 h. For $C_{\text{v}}^{\rho }$ , we find a quartett-like structure peaking at around 24 h and with less pronounced contributions at 21, 26, and 29 h. For both centrality estimates, we observe additional contributions with a period length centered around 12 h (again as triplett-/quartett-like structure), and the impact of other ultradian rhythms appears negligible. The impact of the circadian rhythm on temporal changes of centrality of the LF vertex is stronger pronounced for $C_{\text{v}}^{R}$ than for $C_{\text{v}}^{\rho }$ . We observe again triplett-/quartett-like structures with period lengths that compare to the ones seen for the RMTL vertex. The impact of other ultradian rhythms, including contributions at around 12 h, appears negligible. Within the circadian cycle, particularly $C_{\text{v}}^{R}$ from both vertices exhibits stronger fluctuations (reaching up to 20%) from noon until evening, while the fluctuations of $C_{\text{v}}^{\rho }$ amount to only a few percent. Geier and Lehnertz (2017) reported comparable observations for other vertex centralities of weighted evolving brain networks derived from continuous, long-term, multichannel iEEG data recorded from a larger group of epilepsy patients.

For temporal changes of edge centrality ( $C_{\text{e}}^{R}$ and $C_{\text{e}}^{\rho }$ ), we observe a strong impact of the circadian rhythm for the edge connecting the right mesial temporal lobe and with the left frontal area. For the edge connecting vertices within the frontal area, the rhythm’s impact is strongly pronounced for $C_{\text{e}}^{\rho }$ and barely detectable for $C_{\text{e}}^{R}$ . If detectable, we find triplett-like structures with a period length of the main peak at around 24 h as well as less pronounced contributions around 21 h and around 28 h. For the RMTL—LF edge, there is an additional contribution with a period length at around 12 h (mostly triplett-like structure), and the impact of other ultradian rhythms appears negligible. A 12 h contribution is barely detectable for $C_{\text{e}}^{R}$ of the LF—LF edge, and for $C_{\text{e}}^{\rho }$ of that edge, we find comparable though less pronounced contributions at period lengths around 8 and 12 h. The impact of other ultradian rhythms appears negligible also for this edge. Within the circadian cycle, fluctuations of $C_{\text{e}}^{R}$ of the RMTL—LF edge resemble the ones seen for $C_{\text{v}}^{R}$ of the RMTL vertex, albeit with higher amplitudes (reaching up to 40%). Fluctuations of $C_{\text{e}}^{R}$ of the LF—LF edge amount to only a few percent. Interestingly, for $C_{\text{e}}^{\rho }$ we observe more pronounced fluctuations for the LF—LF edge (reaching up to 20%), while the ones of the RMTL—LF edge amount to only a few percent. As with the clustering coefficient, we note that the temporal fluctuations of the centrality of vertices and edges of the nEEG-based and iEEG-based evolving brain networks comply with a power-law with a scaling exponent *γ* in the range $\gamma \in \left[0.59,1.21\right]$ ). We are not aware of previous reports on such an observation.

Not taking into account dependencies of network characteristics on biological rhythms can lead to erroneous statements about evolving functional brain networks.

## 3 Conclusion

We illustrated the impact of various biological rhythms on temporal changes of characteristics of human brain dynamics at the example of multiday, multichannel non-invasive and invasive EEG recordings from two subjects. We here considered statistical moments and interaction properties of multichannel brain dynamics as well as local and global characteristics of EEG-derived evolving functional brain networks.

We observed the circadian rhythm (with a period length around 24 h) to exert the strongest influence on almost all investigated characteristics. Its impact varied locally, and for characteristics of functional brain networks its influence strongly depended on the approach used to derive networks. For some characteristics, we observed triplett- and/or quartett-like structures accompanying the circadian peak in the respective periodograms. This points to a time-dependent period length of this biological rhythm that is captured by some though not all characteristics. We also observed various ultradian rhythms to impact on temporal changes of the investigated characteristics. Period lengths of these rhythms ranged from 1 to 12 h, and their impact varied locally and strongly differed for the investigated characteristics.

So far, only a few studies related time-dependent fluctuations of EEG signal characteristics (other than those related to power spectral density estimates) to biological rhythms. Ngamga et al. (2016) observed various recurrence-quantification-analysis-based complexity measures for continuous multiday, multichannel invasive EEG recordings from five subjects with epilepsy to fluctuate differently within the circadian cycle, and fluctuations differed for the investigated brain regions. Croce et al. (2018) reported various fractal characteristics of non-invasive EEG recordings from 21 healthy volunteers to be modulated by the circadian rhythm. Modulations varied locally and paralleled changes in alertness and performance. Wilkat et al. (2019) reported the circadian rhythm and ultradian rhythms with period length larger than 4 h to strongly impact on lag-1 autocorrelation and the (unbiased sample) variance of continuous multiday, multichannel invasive EEG recordings from 28 subjects with epilepsy. Since these characteristics are often used in studies that aim at identifying generic early warning signals for critical transitions (e.g., precursors of epileptic seizures), omitting their dependence on biological rhythm renders the reliability of these characteristics problematic. Kurth et al. (2021) reported stochastic qualifiers of brain dynamics to be strongly affected by rhythms acting on time scales that range from hours to days. These findings indicate that biological rhythms even impact on the choice of the stochastic model that may better describe brain dynamics depending on time of day. Kreuz et al. (2004), Porz et al. (2014), and Lehnertz et al. (2017) reported various phase-based estimators for the strength of interactions to be differentially influenced by ultradian and circadian rhythms. Likewise, only a few studies made use of continuous multiday, multichannel EEG recordings from larger groups of subjects with epilepsy to demonstrate fluctuations of local and global characteristics of EEG-derived evolving functional brain networks that can be related to various biological rhythms (Kuhnert et al., 2010; Geier et al., 2015; Geier and Lehnertz, 2017; Lehnertz et al., 2017; Rings et al., 2019).

We are not aware of studies that investigated the impact of infradian rhythms (circaseptan, circatrigintan, or circannual rhythms) on temporal changes of characteristics of human brain dynamics. Nevertheless, when considering time scales of years, a number of studies reported on—at times nontrivial—age-dependencies of characteristics of brain dynamics (see, e.g. (Lindsley, 1939; Duffy et al., 1984; Dustman et al., 1985, 1993; Klass and Brenner, 1995; Anokhin et al., 1996; Polich, 1997; Stam, 2005; Fernández et al., 2012; McIntosh et al., 2014; Sleimen-Malkoun et al., 2015; Zappasodi et al., 2015; Ishii et al., 2017; Knyazeva et al., 2018) and more recently of characteristics of EEG-derived functional brain networks (see, e.g. (Vecchio et al., 2017; Nobukawa et al., 2019; Helmstaedter et al., 2021). In addition to the impact of the various biological rhythms, such age-dependencies would need to be taken into account in order to avoid erroneous statements about brain dynamics and about evolving functional brain networks.

## Data Availability

The original contributions presented in the study are included in the article/supplementary files, further inquiries can be directed to the corresponding author.
